# New extralimital breeding records of saltmarsh sparrows (*Ammospiza caudacuta*) and Nelson's sparrows (*Ammospiza nelsoni*) and their implications

**DOI:** 10.1002/ece3.10532

**Published:** 2023-09-19

**Authors:** Katharine J. Ruskin, Jonathan D. Clark, Alice Hotopp, Adrienne I. Kovach, Nicole A. Guido, Dean L. Hernandez, Colin Peña, Samantha N. Webb, W. Gregory Shriver

**Affiliations:** ^1^ School of Biology and Ecology, Climate Change Institute University of Maine Orono Maine USA; ^2^ Department of Natural Resources and the Environment University of New Hampshire Durham New Hampshire USA; ^3^ Department of Entomology and Wildlife Ecology University of Delaware Newark Delaware USA

**Keywords:** *Ammospiza caudacuta*, *Ammospiza nelsoni*, range expansion, sharp‐tailed sparrows, tidal marsh

## Abstract

Saltmarsh (*Ammospiza caudacuta*) and Nelson's (*A. nelsoni*) sparrows are sister taxa that breed in tidal marshes along the coast of the Northeastern United States and Canada. The Saltmarsh Sparrow breeds from mid‐coast Maine south to Virginia, while the Acadian Nelson's Sparrow breeds from the Canadian maritime provinces south to northern Massachusetts. Here, we present three extralimital observations of breeding Saltmarsh (*n* = 2) and Nelson's (*n* = 1) sparrows. In 2021 and 2022, we observed Saltmarsh Sparrow females attending nests at Mendall Marsh, ME, and Milbridge, ME, respectively, approximately 60 and 110 km beyond the documented northern extent of the Saltmarsh Sparrow breeding range. In 2022, we observed a breeding‐condition male Nelson's sparrow singing in the upriver portion of a marsh on Cape Cod, Massachusetts, approximately 115 km beyond the previously documented southern extent of the Nelson's Sparrow breeding range. We confirmed morphological species identification using a panel of microsatellite DNA loci. Due to both the well‐documented population declines of these species in the region and the intensity of sampling effort undertaken in recent years, we suggest that these observations likely are not indicative of range expansion. However, they do indicate that these 2 taxa have the capacity to use and successfully reproduce in marshes well beyond their established breeding limits. Our findings provide novel insight into the potential for these taxa to occur and successfully breed outside their documented breeding ranges. Given increased interest in their conservation, these results support the idea that management actions aimed at creating or maintaining nesting habitat across both species ranges could benefit both taxa.

## Graphical abstract

Saltmarsh (*Ammospiza caudacuta*) and Nelson's (*A. nelsoni*) sparrows are sister taxa that breed in tidal marshes along the coast of the Northeastern United States and Canada. Here, we present three extralimital observations of breeding Saltmarsh (*n* = 2) and Nelson's (*n* = 1) sparrows. In 2021 and 2022, we observed Saltmarsh Sparrow females attending nests at Mendall Marsh, ME and Milbridge, ME, respectively, approximately 60 and 110 km beyond the documented northern extent of the Saltmarsh Sparrow breeding range. Due to both the well‐documented population declines of these species in the region and the intensity of sampling effort undertaken in recent years, we suggest that unassisted range expansion is unlikely but they do indicate that these 2 taxa have the capacity to use and successfully reproduce in marshes well beyond their established breeding limits.

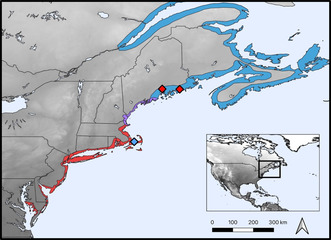

## 1 INTRODUCTION

**FIGURE 1:**
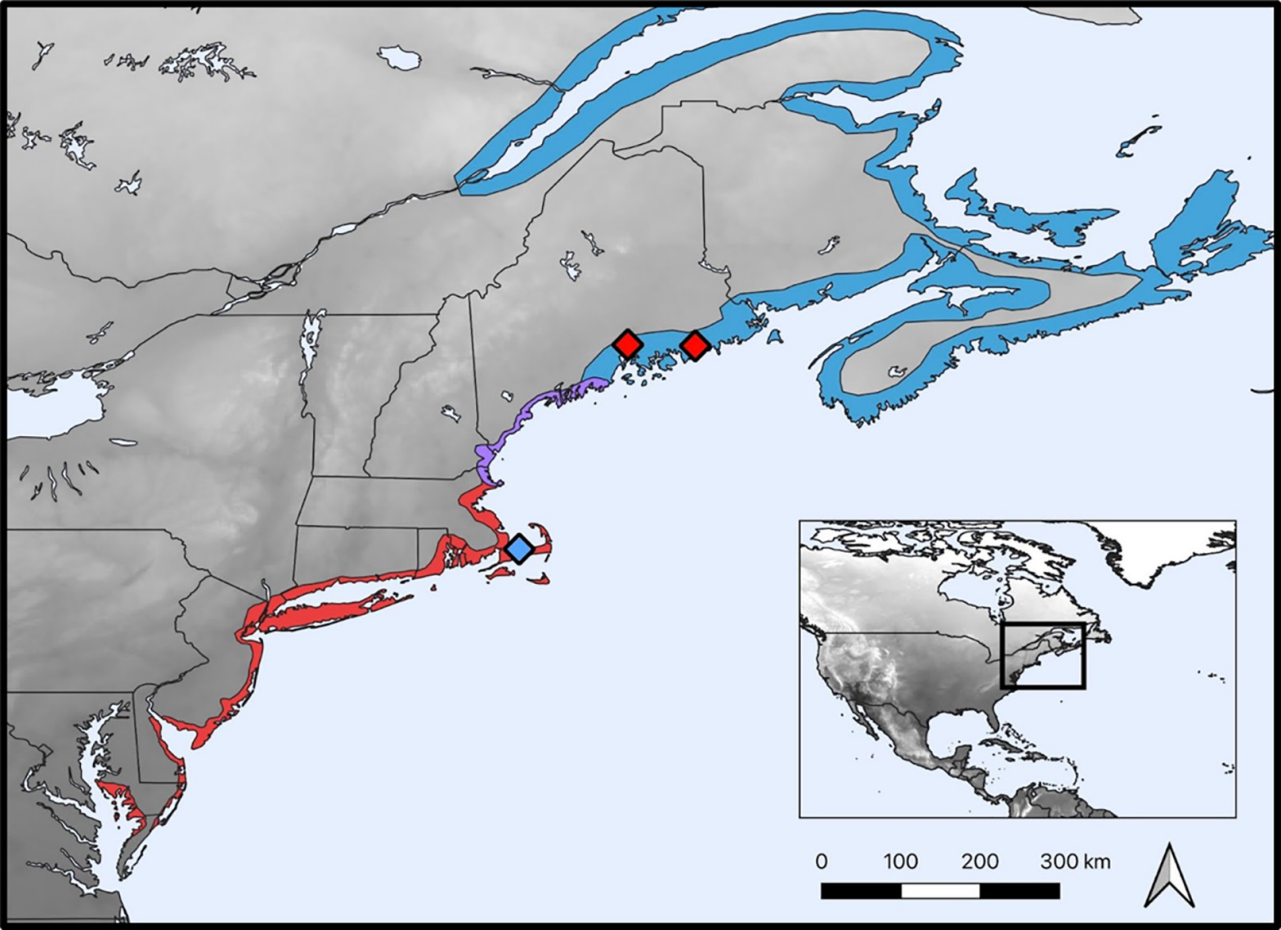
Map of the northeastern United States showing locations of extralimital breeding observations (diamonds). Blue area represents the allopatric breeding range of the Acadian Nelson's Sparrow; red area represents the allopatric breeding range of the Saltmarsh Sparrow; purple area is the established zone of sympatry.

The Saltmarsh Sparrow (*Ammospiza caudacuta*) and Acadian subspecies of the Nelson's Sparrow (*A. nelsoni subvirgata*) are tidal marsh specialists that breed in a narrow ribbon of tidal marsh habitat along the Atlantic coast of the northeastern U.S. and southern Canadian maritime provinces (Figure 1). Prior to 1995, Saltmarsh and Nelson's sparrows were considered a single species, the Sharp‐tailed Sparrow, and were separated subsequently due to genetic and morphological evidence (American Ornithologists' Union, 1995; Rising & Avise, 1993). These sister taxa are still collectively referred to as Sharp‐tailed Sparrows today. The Saltmarsh Sparrow breeding range spans from mid‐coast Maine (Knox County, ME) south to Virginia on the Delmarva Peninsula (Accomack County, VA; Greenlaw et al., 2020). The breeding range of the Acadian Nelson's Sparrow spans the Gulf of St. Lawrence in eastern Quebec south to Plum Island Sound in northern Massachusetts (Hodgman et al., 2002; Shriver et al., 2020). Saltmarsh Sparrows occur exclusively in tidal marshes, while Acadian Nelson's Sparrows are known to use other habitats in addition to tidal marshes—specifically, brackish and freshwater marshes and upland grasslands adjacent to tidal marshes, such as hayfields (Nocera et al., 2007).

Notably, Saltmarsh and Acadian Nelson's sparrows hybridize where their ranges overlap from Plum Island Sound, MA north to Weskeag Marsh, ME (Hodgman et al., 2002, Walsh, Shriver, et al., 2016; Figure 1). In this hybrid zone, both taxa exhibit a range of intermediate phenotypes in morphology and plumage pigmentation (Shriver et al., 2005; Walsh et al., 2015). However, first‐ and second‐generation hybrids are uncommon (Maxwell et al., 2021; Walsh et al., 2015; Walsh, Olsen, et al., 2016). Outside of the hybrid zone, there are detectable signals of interspecific gene flow as far north as the Quoddy Narrows in Lubec, ME and as far south as the coast of Massachusetts Bay (Walsh, Shriver, et al., 2016) or possibly coastal Connecticut (J. Walsh, Cornell Lab of Ornithology, 2023, unpublished data).

The ranges of Saltmarsh and Acadian Nelson's sparrows within the United States have been well studied in the past two decades, including multiple surveying efforts that have led to updated range maps for these species. For the Saltmarsh Sparrow, range contraction over the past century is evident near the southern range boundary (Greenlaw et al., 2020). The documented northern boundary of Saltmarsh Sparrows has shifted northward (Hodgman et al., 2002; Montagna, 1940). However, paucity of historical data, unclear survey efforts, and the previous conspecific classification of Saltmarsh and Nelson's sparrows confounds the exact extent of the northern range boundary. Researchers have generally concluded that the northern extent of the Saltmarsh Sparrow breeding range, Weskeag Marsh (Thomaston, ME, 44°4′16.32″ N, 69°8′42″ W) (Hodgman et al., 2002), has changed little if at all (Greenlaw et al., 2020). For the Nelson's Sparrow, the breeding range is generally unchanged in recent decades (Shriver et al., 2020) though some evidence suggests a southerly expansion of Acadian Nelson's Sparrows (Walsh et al., 2017).

The patterns of range shift experienced by these taxa—a contracting range for the Saltmarsh Sparrow and a stable range for the Acadian Nelson's Sparrow—mirror the population trends for these species. Point count surveys from Maine to Virginia demonstrated that Saltmarsh Sparrows declined 9.0% annually between 1998 and 2012 (Correll et al., 2017). The species is expected to be globally extinct by 2060 (Field et al., 2017) and is classified as Endangered by the International Union for Conservation of Nature (IUCN, 2022). In the same geographic range, Acadian Nelson's Sparrows exhibited a 4.2% annual decline between 1998 and 2012, but some subregions exhibited non‐significant and even positive population growth rates (Correll et al., 2017). Long histories of anthropogenic habitat loss and degradation (e.g., changes to marsh hydrology such as tidal restriction and ditching) are listed as threats to both species (Greenlaw et al., 2020; Shriver et al., 2020). The population declines observed since the 1990s have been linked to sea‐level rise for Saltmarsh Sparrows (Field et al., 2017; Ruskin, Etterson, Hodgman, Borowske, Cohen, Elphick, Field, Longenecker, et al., 2017), while the relationship between sea‐level rise and populations of the Acadian Nelson's Sparrow has not been found to be significant (Correll et al., 2017; Shriver et al., 2015).

Given their observed population declines, there is increasing conservation interest in both taxa, and it is of utmost importance to accurately characterize their contemporary breeding ranges. Here, we report three extralimital records of breeding Saltmarsh and Nelson's sparrows. We discuss questions raised by these new breeding records and how they may relate to the conservation of these taxa.

## 2 METHODS

### 2.1 Field surveys

The observations reported here were made as part of a larger, regional effort to survey Saltmarsh and Nelson's sparrows throughout the northeastern United States. Since 2011, the Saltmarsh Habitat and Avian Research Program (SHARP; www.tidalmarshbirds.org) has conducted intensive demographic sampling at 40 tidal marsh study plots from Maine to Virginia, ranging from 6 to 21 plots per year. At each intensive demographic plot, we used mist‐netting to capture sparrows for survival estimation (Field et al., 2018) and nest searching and monitoring to estimate reproductive success (Ruskin, Etterson, Hodgman, Borowske, Cohen, Elphick, Field, Kern, et al., 2017). We located each demographic study plot in tidal marsh habitat dominated by *Spartina patens*. Study plots were approximately 5–15 hectares and we visited each once per 2–4 days, with nest searching across the entire plot occurring at least once per week. Nests for both species are typically found using behavioral cues, particularly flushes of females from nests (generally 5–15 m from the observer) while walking systematically through the marsh. From 2011 to 2022 and spanning a total range of 575–992 km each year, we have observed 1252 nests and 22,459 captures of 13,420 individual Saltmarsh and Nelson's sparrows (SHARP, 2011–2022, unpublished data).

In addition, SHARP crews conducted opportunistic demographic surveying across this range in multiple breeding seasons at additional tidal marshes over the last decade: 2012–2013 (*n* = 34 sites, ME to CT, Walsh et al., 2016,c); 2015–2016 (*n* = 49 sites, ME to VA, Conway, 2019); 2021–2022 (*n* = 36 sites, ME to NC, J. Clark, University of New Hampshire, 2021–2022, unpublished data); 2019, 2021–2022 (*n* = 91 sites, ME to VA, Sanchez Jr., 2023). These additional surveys focused on adult captures, and therefore involved mist‐netting in high marsh habitats in areas of high tidal marsh bird concentration. In contrast to intensive demographic surveys, opportunistic demographic surveys involved little or no nest searching and monitoring and occurred at each site on 1–2 days per breeding season. Over this 10‐year period, these surveys yielded approximately 5000 captures but fewer than 70 nests, as mist‐netting was the primary focus of these studies and nests were only recorded as opportunistically encountered. Though the larger context of SHARP's regional data collection effort is important for the interpretation of the extralimital records reported here, the three records we report here were observed during intensive demographic sampling at two study plots for both Saltmarsh Sparrow nesting records and during opportunistic demographic sampling from ME to NC for the Nelson's Sparrow record.

We identified species based on plumage traits observed in the field and verified species using a plumage index developed by Shriver et al. (2005) and genotype. For plumage index, we used photographs to score individuals for 13 traits. Briefly, we scored each trait from 1 to 5 with lower numbers representing features indicative of Nelson's Sparrow and higher numbers indicative of Saltmarsh Sparrow. Summed plumage scores range from 13 (pure Nelson's Sparrow) to 65 (pure Saltmarsh Sparrow) (Shriver et al., 2005). We used the thresholds defined by Walsh et al. (2015) to classify individuals, with scores of 13–31 for Nelson's Sparrow, 46–65 for Saltmarsh Sparrow (intermediate scores of 32–45 are indicative of hybrids).

To confirm the morphological species assignment for each of these three birds, we genotyped a panel of microsatellite DNA markers previously shown to differentiate the species and characterize introgression of individuals in the Saltmarsh–Nelson's hybrid zone (Kovach et al., 2015; Walsh et al., 2015). We followed the protocols of Walsh et al. (2015) to amplify 20 diagnostic microsatellite markers for the three extralimital birds and a reference set of allopatric Saltmarsh (*n* = 15; from Connecticut, Rhode Island, and Massachusetts) and Nelson's sparrows (*n* = 19; from Maine). The resulting amplicons were sent to the Keck DNA Sequencing Lab at the Yale School of Medicine for characterization of fragment length polymorphisms using an Applied Biosystems 3130 Series Genetic Analyzer. Alleles were scored manually with PeakScanner software. We then used genotype data to calculate hybrid index (0 representing pure Nelson's Sparrow and 1 representing pure Saltmarsh Sparrow) and interspecific heterozygosity for each individual with the R package INTROGRESS (Gompert & Buerkle, 2010). We assigned individuals to genotypic classes following Walsh, Rowe, et al. (2016), such that a hybrid index of 0–0.05 (Acadian Nelson's Sparrow) or 0.95–1.0 (Saltmarsh Sparrow) was indicative of a genotypically pure individual, an intermediate hybrid index value (0.25–0.75) and high interspecific heterozygosity (>0.3) was indicative of a recent generation hybrid (F1 or F2), and hybrid indices of 0.05–0.25 or 0.75–0.95 and low interspecific heterozygosity (<0.3) were indicative of backcrossed Nelson's Sparrows and back‐crossed Saltmarsh Sparrows, respectively.

## 3 RESULTS

### 3.1 Extralimital breeding observations

#### 3.1.1 Saltmarsh sparrow; Mendall marsh, Waldo County, Maine

**FIGURE 2:**
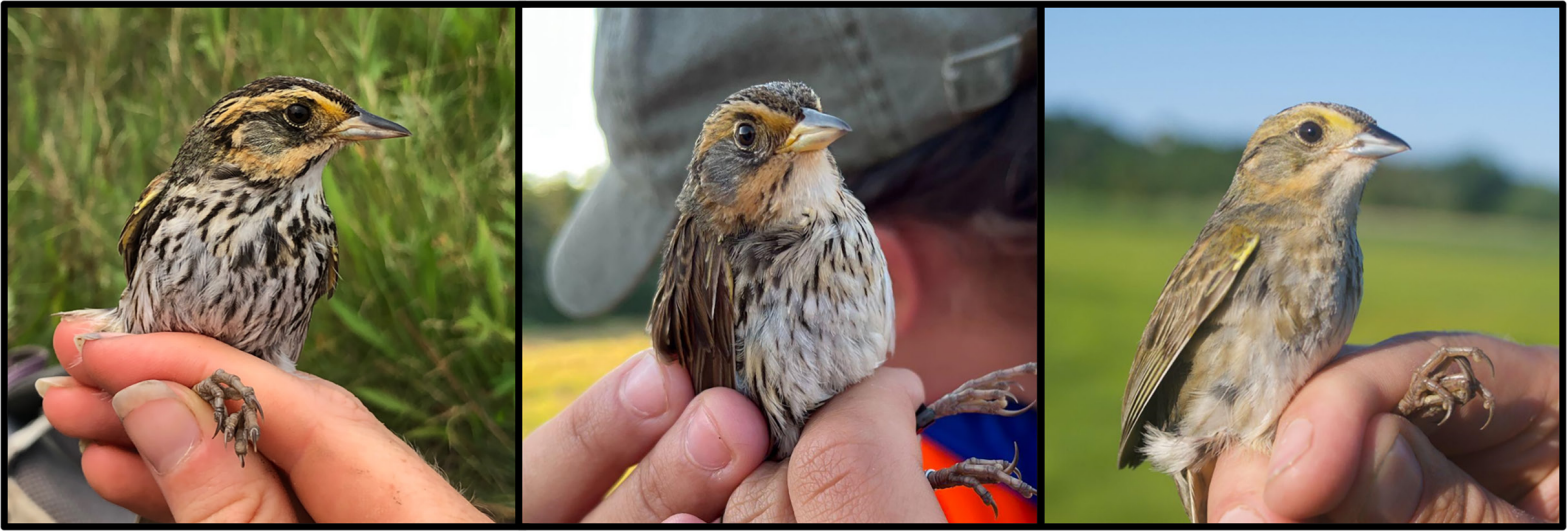
Photos of the Saltmarsh Sparrow females from Mendall Marsh, ME (left) and Milbridge, ME (center), and of the male Nelson's Sparrow from Cape Cod, MA (right).

On 20 July 2021, during systematic nest searching at known breeding sites of Nelson's Sparrows at Mendall Marsh in Waldo County, ME (44°35′20.4″ N, 68°51′32.4″ W), we found a nest via a female flush (at 10 m distance) that contained four eggs. The nest had a partially woven canopy and the square meter around the nest was dominated by *Spartina patens* and *Solidago sempervirens*. We conducted targeted mist netting at the nest immediately after discovery and captured the attending female approximately 1 h after we discovered the nest. We identified the female as a Saltmarsh Sparrow based on plumage characteristics (plumage score from photographs = 55 out of a range of 13–65, using the index of Shriver et al., 2005; Figure 2). We banded the bird with a uniquely numbered aluminum band issued by the U.S. Geological Survey (USGS; 2821‐27744). We revisited the nest every 3 days to track its fate, which we classified as partially failed due to flooding on 23 July 2021 and completely destroyed due to flooding on 26 July 2021, following the monthly high tide which often floods Saltmarsh and Nelson's sparrow nests (Bayard & Elphick, 2011; Ruskin, Etterson, Hodgman, Borowske, Cohen, Elphick, Field, Longenecker, et al., 2017; Shriver et al., 2005). At the time of observation, this nest at Mendall Marsh was the northernmost documented nesting for Saltmarsh Sparrows.

On 29 July 2021, we discovered a new nest with three eggs that we hypothesize was built by the same Saltmarsh Sparrow female because (1) we identified the attending female as a Saltmarsh Sparrow with binoculars, (2) the new nest was within 30 m of the first nest, and (3) the composition of the surrounding vegetation of the second nest site was similar to the first. We were unable to confirm whether the nest belonged to the Saltmarsh Sparrow female because we failed to capture the attending female on 29 July 2021, when she was not visiting the nest often, likely because she was laying eggs at this time and not yet incubating. At the next visit on 2 August 2021, the nest was depredated.

#### 3.1.2 Saltmarsh sparrow; Milbridge, Washington County, Maine

On 7 July 2022, during systematic nest searching at known breeding sites of Nelson's Sparrows along the Narraguagus River near Milbridge in Washington County, ME (44°34′19.2″ N, 67°54′50.4″ W), we found a nest via female flush (at 3 m distance) that contained three eggs. The nest had a partially woven canopy and the square meter around the nest was dominated by *Spartina patens* and *Carex paleacea*. We conducted targeted mist netting at the nest on 11 July 2022 and captured the attending female. We identified the female as a Saltmarsh Sparrow based on plumage characteristics (plumage score from photographs = 57; Figure 2). We banded the bird with a uniquely numbered aluminum USGS band (2881‐26898) and a Passive Integrated Transponder (PIT) tag (A.R. Kocek, State University of New York Environmental Science and Forestry, 2022, unpublished data). For other research questions, females at some SHARP study plots were banded with PIT tags to allow association with future nesting attempts without capture. We revisited the nest every 2 days to track its fate. On 13 July 2022, following the monthly high tide, we observed that the nest failed due to flooding. Despite nest failure, we captured the Saltmarsh Sparrow female again in the same area of the marsh on 25 July 2022 during a general mist‐netting effort.

On 1 August 2022, we found a nest in a similar area of the marsh with four nestlings aged as 2 days old. The nest had no woven canopy and was located in *Spartina patens* and *Carex paleacea*. We deployed an RFID reader at the nest and detected the RFID number of the Saltmarsh Sparrow female, confirming a second nesting attempt. Again, we revisited the nest every 2–3 days, and on 6 August 2022, we banded all four of the nestlings (aged 6 days) with uniquely numbered aluminum USGS bands (2881‐26718 to 2881‐26721). On 9 August 2022, we observed the Saltmarsh Sparrow regularly delivering food to the nest which would have contained 9‐day‐old nestlings. On the following visit on 12 August 2022, the nest was empty with characteristic signs of a successfully fledged nest (feces in and around nest bowl, no sign of disturbance), making this nest the northernmost documented record of nesting and fledging for Saltmarsh Sparrows.

#### 3.1.3 Nelson's sparrow; Cape Cod, Barnstable County, Massachusetts

On 11 July 2022, we deployed a mist net array to capture Saltmarsh Sparrows in the tidal marsh at Sandy Neck Beach Park in Barnstable, MA (41°43′37.2″ N, 70°22′48″ W). We detected a Nelson's Sparrow‐type song several times near the array in the early morning. Therefore, we played Nelson's Sparrow vocalizations from a speaker placed near the net to elicit a territorial response from the singing individual, causing a sparrow to become agitated near the net. Several minutes later, this sparrow was captured in the array and identified as an adult Acadian Nelson's Sparrow based on plumage characteristics (plumage score from photographs = 26; Figure 2). We identified the individual as a male in breeding condition based on the presence of a prominent cloacal protuberance. The individual lacked any evidence of molt that would indicate it had begun migration. We banded the bird with a uniquely numbered aluminum USGS band (2811‐83672). In addition to the Acadian Nelson's Sparrow male, we captured 13 other birds at the site that morning (six female and seven male Saltmarsh Sparrow adults, all in breeding condition).

### 3.2 Genetic species identification

The Acadian Nelson's Sparrow from Cape Cod exhibited genotypes consistent with a genetically pure Nelson's Sparrow (hybrid index = 0.03, interspecific heterozygosity = 0.10). The two Saltmarsh Sparrows from Maine each exhibited genotypes expected for a backcrossed Saltmarsh Sparrow, suggesting they had some recent Nelson's sparrow ancestry but were not themselves hybrids. (Mendall Marsh: hybrid index = 0.82, interspecific heterozygosity = 0.30; Milbridge: hybrid index = 0.80, interspecific heterozygosity = 0.20). The values of hybrid index and interspecific heterozygosity for both Saltmarsh Sparrow individuals are common for Saltmarsh Sparrows from the hybrid zone (Maxwell et al., 2021).

## 4 DISCUSSION

We report three extralimital breeding observations, which prompt a number of important questions. First: *are the Saltmarsh or Nelson's sparrow ranges expanding?* This question is of critical importance given the declines observed in both Saltmarsh and Acadian Nelson's sparrows (Correll et al., 2017; Wiest et al., 2016). SHARP has undertaken extensive demographic and surveying effort since 2011, and the three extralimital observations reported here are the only deviations from the ranges as described by Hodgman et al. (2002) we have observed. Given the amount of search effort and the rarity of extralimital encounters we have observed, we hypothesize that the records we report here likely represent very rare occurrences that we observed as a result of intensive surveying.

Broadly, extralimital records for birds are relatively common, especially among young individuals (Harris et al., 2002; Selonen & Hanski, 2006; Tonelli et al., 2023; Towers et al., 2022; Zawadzki et al., 2019). Saltmarsh and Nelson's sparrows cannot be classified beyond hatching year or after hatching year by plumage because they exhibit complex alternate strategy for molting, including partial‐to‐incomplete preformative and prealternate molts (Greenlaw et al., 2020; Shriver et al., 2020). Therefore, we cannot determine whether the individuals we observed breeding extralimitally were young (only 1 year old) and inexperienced (first time breeders).

Regarding the extralimital male Acadian Nelson's Sparrow, a singing male is not necessarily a strong indicator of breeding range expansion. For example, in our own surveys, we have occasionally observed a lone male Seaside Sparrow (*A. maritima*)—another tidal marsh obligate songbird—singing in marshes in Scarborough, ME, and Phippsburg, ME, approximately 80 and 120 km north of their established breeding range, respectively. Further, male Saltmarsh Sparrows have been documented singing in high abundance at a small Connecticut marsh with no females or nesting detected (C.S. Elphick, University of Connecticut, 2023, personal communication), demonstrating that Saltmarsh Sparrow males will occupy and sing at sites where no breeding is occurring. Nevertheless, this male Nelson's Sparrow was singing in a marsh with an abundance of female Saltmarsh Sparrows, and given that these species readily hybridize (Greenlaw et al., 2020, Shriver et al., 2020), it is possible that this individual sired offspring by local female Saltmarsh Sparrows. Moreover, previous research has detected signals of interspecific gene flow south of the hybrid zone extending to Cape Cod Bay (Walsh, Shriver, et al., 2016) and possibly as far south as coastal Connecticut (J. Walsh, Cornell Lab of Ornithology, 2023, unpublished data). Walsh et al. (2017) examined both genetic and distributional surveys and found some evidence of southerly range expansion of the Nelson's Sparrow. In addition to increased introgression, they observed an increase in the proportion of Nelson's Sparrows relative to Saltmarsh Sparrows in the hybrid zone in 2012 as compared to 1998, as well as detection of Nelson's Sparrows at two sites south of the hybrid zone in 2012. Increased interspecific gene flow within and outside of the hybrid zone could be the result of stepwise gene flow from hybrid zone sites, occasional extralimital breeders, or a combination thereof.

For Saltmarsh Sparrows, we have now observed three nests built outside of the established breeding range by two different females. One nest detected on the Narraguagus River, ME fledged young, confirming successful breeding by a Saltmarsh Sparrow at this site previously considered to support only Nelson's Sparrows. Saltmarsh Sparrows exhibit natal philopatry (DiQuinzio et al., 2001; Greenlaw et al., 2020), which could facilitate recruitment of breeding adults that would add Saltmarsh Sparrow alleles to the population if the nestlings survive and return to the Narraguagus River to breed. Therefore, we recommend future monitoring at the Narraguagus River, Mendall Marsh, and other nearby sites once thought to be occupied by Nelson's Sparrows only, to (1) assess whether the nesting females we observed or their offspring return in subsequent years, (2) determine whether other Saltmarsh Sparrow individuals become established breeders at these sites, and (3) identify other nearby sites where Saltmarsh Sparrows may be breeding.

Importantly, even if these observations represent expansion of the Saltmarsh and Nelson's sparrow breeding ranges, the expansions would be smaller than the range contraction experienced by Saltmarsh Sparrows at their southern range boundary (Greenlaw et al., 2020). Additionally, potential range expansions are further offset by the large declines exhibited by these species. Saltmarsh Sparrows are declining 9.0% annually and are expected to be extinct by mid‐century, while Acadian Nelson's Sparrows in this region are declining 4.2% annually (Correll et al., 2017). For Saltmarsh Sparrows, observed declines are driven by low nest survival associated with sea‐level rise (Field et al., 2017). For Acadian Nelson's Sparrows, population trends are less clear. Nelson's Sparrow population trends are less well studied (Shriver et al., 2020) and mixed (declining, stable, or positive) depending on scale and region (Correll et al., 2017; Shriver et al., 2020, 2015). As tidal marsh specialists, sea‐level rise and other drivers of saltmarsh degradation likely threaten Nelson's Sparrow populations (Correll et al., 2017), but sea‐level rise has not yet been associated with their observed population trends (Correll et al., 2017; Shriver et al., 2015).

A Saltmarsh Sparrow successfully fledging young in eastern Maine, 110 km beyond the reported northern range boundary raises a second important question: *should marshes in eastern Maine be considered potential Saltmarsh Sparrow habitat?* The Narraguagus River record we report demonstrates reproductive success, indicating that the habitat is suitable for successful Saltmarsh Sparrow breeding as defined by fledging. Again, further investigation is needed to determine whether the marshes can support breeding to the point of recruiting Saltmarsh Sparrow nestlings into adulthood. Given the rarity with which we observed Saltmarsh Sparrows breeding extralimitally, we conclude that it is unlikely for this species to establish breeding populations in eastern Maine without intervention. Nonetheless, our observation that this region provides viable breeding habitat for Saltmarsh Sparrows opens the door for managers to consider using range expansion as a conservation tool. At minimum, the observations we report here reveal that eastern Maine marshes can support breeding for both species. Therefore, actions focused on benefiting Nelson's Sparrows (e.g., restoration or creation of *S. patens*‐dominated habitats in eastern Maine) have the potential to simultaneously benefit Saltmarsh Sparrows. Given the rapid decline of Saltmarsh Sparrows, considering saltmarshes along an additional 110 km of Maine's coastline as viable habitat for the species is an asset for conservation. Further, sites in the north of the Saltmarsh Sparrow range exhibit relatively high rates of nest survival compared to others in the range (Field et al., 2017; Ruskin, Etterson, Hodgman, Borowske, Cohen, Elphick, Field, Kern, et al., 2017).

Until recently, most conservation actions for tidal marsh birds have consisted of habitat preservation. The majority of tidal marshes in the northeastern United States are under conservation ownership or easement (57%; USGS PAD, 2022), and the Clean Water Act (1972) prevents any development in wetlands including tidal marshes without extensive permitting. These protections do not guard against sea‐level rise, however, and population declines in Saltmarsh and Acadian Nelson's sparrows persist. The conservation community has recently begun to consider and implement more active management strategies to benefit Saltmarsh Sparrows and other tidal marsh birds. These include marsh elevation enhancement, hydrologic restoration, manual elevation of Saltmarsh Sparrow nests, creating floating islands of marsh habitat, building mounds of sediment for elevated nesting habitat, controlling tide levels, and tree‐cutting to facilitate marsh migration (Hartley & Weldon, 2020). Cook et al. (2021) reported that within‐marsh translocation of Saltmarsh Sparrow nestlings has been successful. A documented positive response to translocation combined with the natal philopatry observed in these species (DiQuinzio et al., 2001), means that managers may be able to establish Saltmarsh Sparrow populations in marshes that are north of the current range limit. Therefore, assisted migration can be considered among the portfolio of interventions aimed at preserving tidal marsh bird populations in the face of sea‐level rise.

## AUTHOR CONTRIBUTIONS

**Katharine J. Ruskin:** Conceptualization (lead); data curation (equal); writing – original draft (lead); writing – review and editing (equal). **Jonathan D. Clark:** Formal analysis (lead); investigation (equal); visualization (lead); writing – original draft (supporting); writing – review and editing (equal). **Alice Hotopp:** Data curation (equal); investigation (equal); writing – review and editing (equal). **Adrienne I. Kovach:** Funding acquisition (supporting); supervision (equal); writing – review and editing (equal). **Nicole A. Guido:** Investigation (supporting); writing – original draft (supporting); writing – review and editing (equal). **Dean L. Hernandez:** Investigation (supporting); writing – original draft (supporting); writing – review and editing (equal). **Colin Peña:** Investigation (supporting); writing – original draft (supporting); writing – review and editing (equal). **Samantha N. Webb:** Investigation (supporting); writing – original draft (supporting); writing – review and editing (equal). **W. Gregory Shriver:** Funding acquisition (lead); investigation (equal); supervision (equal); writing – original draft (supporting); writing – review and editing (equal).

## CONFLICT OF INTEREST STATEMENT

The authors have no conflict of interest to declare.

## Supporting information


Data S1.

## Data Availability

Genotyping data are included as supplementary files upon acceptance. Sampling locations and morphological data: in text.
